# A Bibliometric and Visualization Analysis of Intermittent Fasting

**DOI:** 10.3389/fpubh.2022.946795

**Published:** 2022-07-06

**Authors:** Shiying Chen, Rui Han, Haitao Liu

**Affiliations:** ^1^College of Physical Education, Henan University, Kaifeng, China; ^2^Research Center for Sports Reform and Development, Henan University, Kaifeng, China

**Keywords:** intermittent fasting, time restricted feeding, CiteSpace, cluster analysis, bibliometric, co-citation analysis

## Abstract

CiteSpace software was utilized to visually analyze the literature on intermittent fasting from Web of Science from 2000 to 2020 in order to reveal the current status, research hotspots and emerging trends of intermittent fasting. The results show that: (1) intermittent fasting research results are increasing year by year; (2) the United States is at the core of this field and has a high influence; (3) intermittent fasting research is mainly concentrated in the fields of nutrition, cell biology and kinesiology, which embodies interdisciplinary characteristics; (4) the literature of Sutton, Mattson and Trepanowski that were published in the same period have the highest co-citation frequencies, however, their research perspectives are quite different, reflecting that the research in this field is still in a state of continuous development; (5) from the perspective of citation bursts, the evolution of research hotspots in this field in the last 20 years can be divided into 3 stages; (6) the keyword timeline mapping shows that time restricted feeding is at the forefront of this research field. This study can help researchers explore the field for the first time to quickly grasp the frontiers and obtain more valuable data, thereby providing facilitation for the follow-up research.

## Introduction

Modern humans are faced with complex health challenges, and the morbidity and mortality of metabolic diseases associated with obesity extremely affect the economy. As current treatments for obesity are limited and offer only modest improvements, it is essential to find a new way to treat obesity (1). Lifestyle changes are more convenient and less costly than pharmacological or surgical approaches. One recently popular dietary strategy, intermittent fasting (IF) that has many potential health benefits is a non-pharmacological strategy for obesity and related diseases (2, 3). Incorporating an IF lifestyle into adult daily life can promote health and reduce the risk of many chronic diseases, especially for those who are overweight and sedentary (4).

ADF regimens generally involve a “feast day” on which food is consumed *ad libitum* that alternates with a “fast day” on which food is withheld or reduced. The feast and fast periods are typically 24 h each, but they may vary.

Intermittent fasting refers to a dietary intervention that alternates between free-feeding and fasting in a continuous cycle (5–8). Most intermittent fasting therapies can be divided into three main categories (9). Alternate day fasting (ADF) programs typically involve a “festival” on which food is consumed arbitrarily, alternating with a “fasting day” in which no food is eaten or reduced on that day (10). Whole-day fasting (WDF) generally consists of one to two non-consecutive full fasting days per week, with free eating the rest of the time (11). Time restricted feeding (TRF) is when food intake is restricted to a specific part of the day and fasting the rest of the time (12). Although there are different forms of intermittent fasting, studies have demonstrated fasting is a sustainable and easy to implement lifestyle with many benefits for the body (13–23). Results from human studies suggest that IF cannot only prevent metabolic syndrome and related diseases, such as diabetes and cardiovascular disease (24–26), but also change body composition, promote cardiometabolic health and increase life expectancy (27–29). However, the mechanism remains unknown. The reasons for the potential effectiveness of IF include gut microbiota, oxidative stress, circadian rhythms, inflammation, insulin, and the brain derived neurotrophic factor (11, 30–33).

In recent years, studies on intermittent fasting have gradually increased, but most of them focus on the effect of intermittent fasting on a particular disease area. With the rapid development of intermittent fasting, we need to systematically understand the development process and research trends in the field, however, there is a lack of bibliometric and visualization analysis article on intermittent fasting. CiteSpace literature visualization and analysis software, which can reflect the research orientation of a field in the form of dynamic mapping (34). Therefore, this paper uses CiteSpace software to visually analyze the literature on intermittent fasting from 2000 to 2020, thus revealing the trends and research hotspots in this field, helping researchers explore this field for the first time to quickly grasp the frontiers of research in this field and obtain more valuable data, and providing new ideas to promote national health research.

## Data Collection and Research Method

### Data Collection

**Figure 1:**
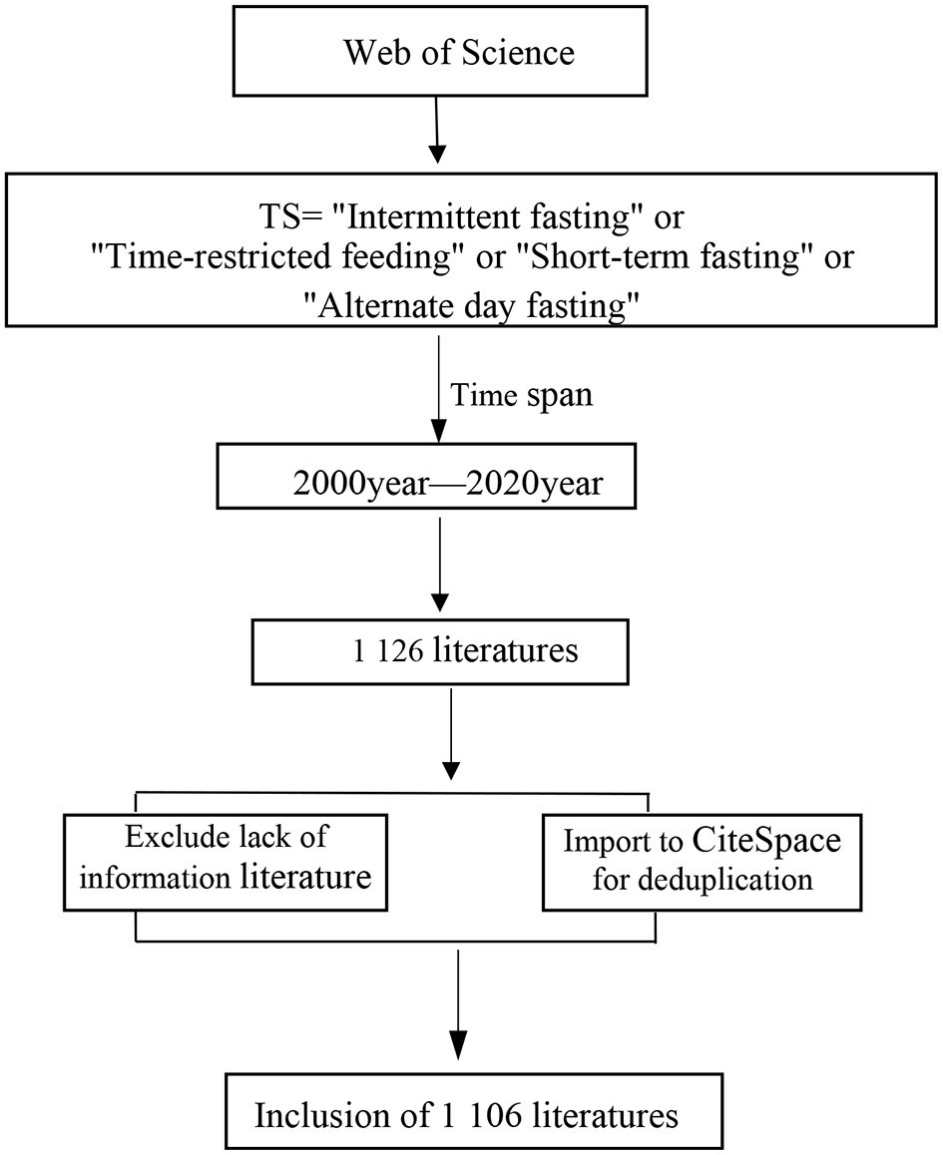
The flow chart of searching papers in databases.

The Web of Science core collection of journal articles represents the highest level of international social science research. When CiteSpace was performed on visual analysis, the Web of Science database can present a better knowledge graph effect (35, 36). Therefore, this study retrieved the required literature from Web of Science Core Collection. As there are many foreign classifications of intermittent fasting, a relatively broad search topic was used to ensure the comprehensiveness of the literature. Using Web of Science as the data source, the search was conducted using “Intermittent fasting,” “Time-restricted feeding,” “short-term fasting,” “alternate day fasting” as the subject terms, and the time span was from 2000 to 2020, a total of 1,126 articles were retrieved. The full information of the literature (including title, abstracts, authors, keywords, document types, journals, year of publications and the cited references) was downloaded and exported to Ref-Works citation format. However, some pieces of literature lacked information, such as authors and references, so these records were excluded. Then, the exported literature information was imported into CiteSpace software for data preprocessing, such as filtering and duplication. Finally, a total of 1,106 effective pieces of literature are obtained (Figure 1).

### Research Methods

CiteSpace software is an important analytical and visualization tool in the field of scientometrics, which can be used to explore the current state of research, research hotspots, evolutionary processes and disciplinary structure of a scientific field, so as to grasp the research direction of institutions and authors and judge the classical literature and related supporting studies (37, 38). The study was conducted using CiteSpace V. In this study, CiteSpace V (version: 5. 5. R2) was used to visualize and analyze the sample literature data in order to obtain the evolutionary relationship between intermittent fasting hotspots and the knowledge base. The processing parameters were set as follows: year interval 2000-2020, time slice of 1 year, country, institution, discipline, author, cited literature, etc. according to the topic of study, and threshold criterion of Top 50.

## Results

### Time Distribution

**Figure 2:**
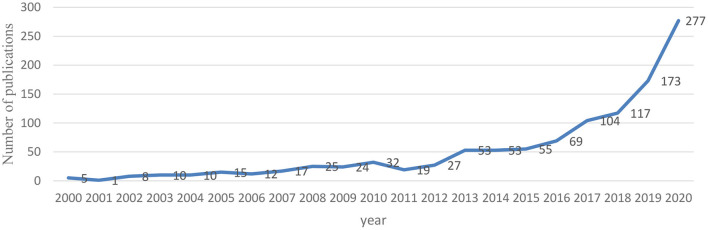
Trends in publications in the field of intermittent fasting from 2000 to 2020.

The number of articles published each year reflects the activity of the field and the importance given to a particular research area (39). The annual distribution of the literature on intermittent fasting over the past 20 years was obtained by counting the volume of literature on intermittent fasting during the period 2000–2020 (Figure 2). It shows that the overall trend of intermittent fasting-related literature is increasing year by year, indicating that scholars are gradually paying more attention to the field of intermittent fasting.

As can be seen from Figure 2, the development of intermittent fasting over the past two decades can be divided into two phases: phase 1 is a steady growth phase (2000–2016) with an annual average volume of 25 articles. Caloric restriction has been shown to have many benefits for the body since as early as 1935 (40). After 2000, a new dietary strategy, intermittent fasting, has been studied and proven to have beneficial effects on health (41, 42). The literature on intermittent fasting in this phase shows a steady increase.

Phase 2 is a period of sustained growth (2017–2020), with an average of 167 publications per year, which is at a high level, indicating the rising research fervor and importance of intermittent fasting. The number of publications exceeds 100 for the first time in 2017 and reaches a 20-year peak in 2020. Mattson's 2017 review of the effects of intermittent fasting on health and disease processes has attracted widespread scholarly attention and driven the rapid development of intermittent fasting (24). Intermittent fasting has become a popular dietary strategy. As people become more concerned about their health and quality of life, diet is a key part of health promotion to drive the development of diet and health-related research (43). Research related to intermittent fasting will continue to receive extensive attention from scholars, and research in this area will be progressively deepened.

### Spatial Distribution

#### Country Distribution

**Figure 3:**
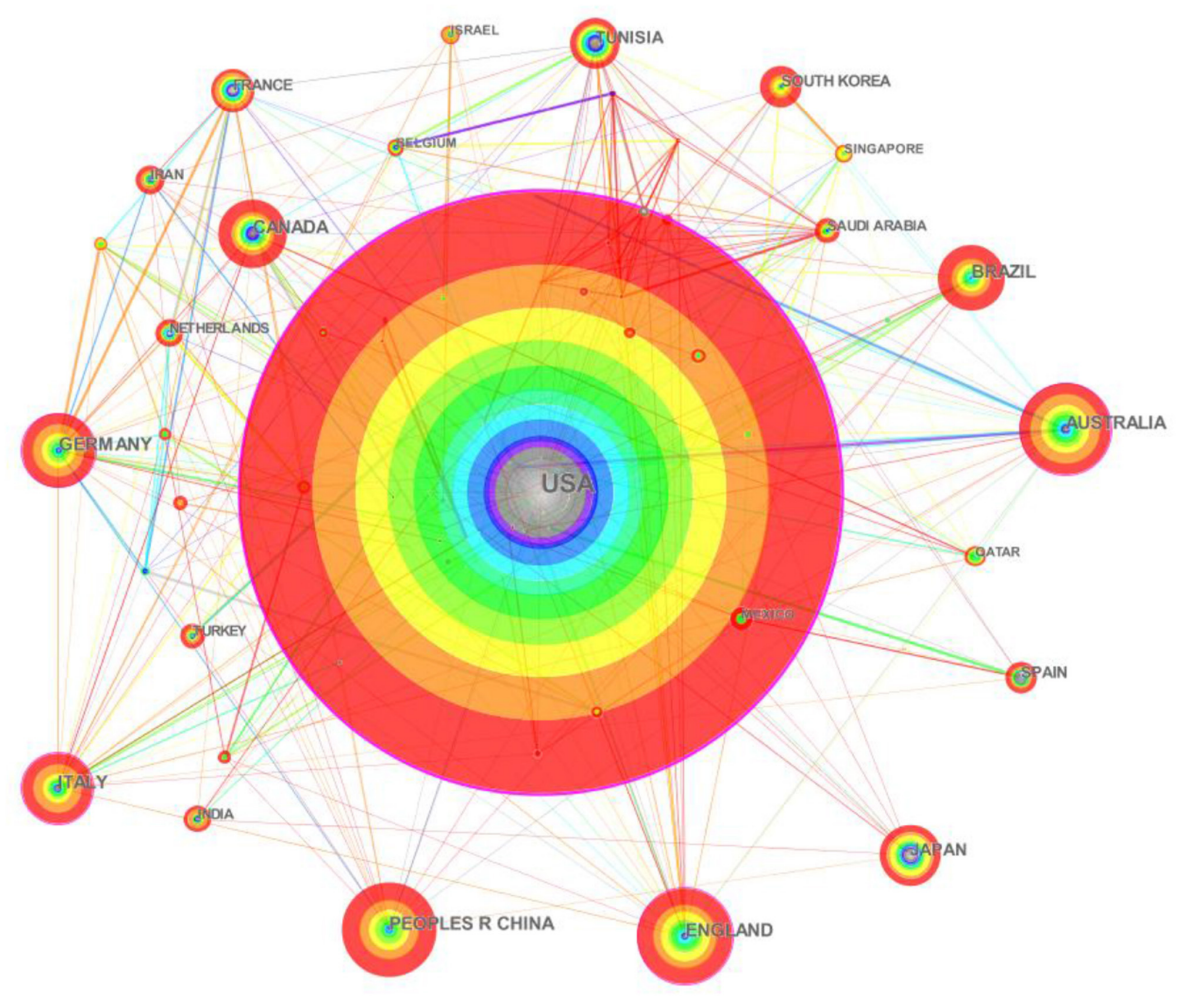
Knowledge map of the co-country/territory collaboration network.

The parameter “Country” was selected in CiteSpace and a knowledge map of the national/regional cooperation networks in the field of intermittent fasting was obtained (Figure 3). Each node represents a country (region), and the size of the node is proportional to the number of articles published; the lines connecting the nodes represent the connections between them, and the thickness of the lines is proportional to the closeness of their connections; the outermost circle represents centrality, which is a measure of the size of the connections in the knowledge graph network, and is the hub of communication with other literature nodes (44). Combining the results of the software analysis, the TOP 10 countries in the field of intermittent fasting were compiled (Table 1).

**Table 1:** Top 10 countries (regions) in terms of number of publications in the field of intermittent fasting research.

**Rank**	**Country**	**Area of affiliation**	**Publications**	**Centrality**
1	USA	North America	424	0.49
2	PEOPLES R CHINA	Asia	70	0.04
3	ENGLAND	Europe	70	0.12
4	AUSTRALIA	Oceania	67	0.15
5	GERMANY	Europe	55	0.13
6	ITALY	Europe	53	0.12
7	CANADA	North America	53	0.06
8	BRAZIL	Asia	50	0.09
9	JAPAN	Asia	48	0.01
10	TUNISIA	Africa	39	0.08

One thousand and one hundred-six English-language articles in this field were from 102 countries (regions). Table 1 presents the top 10 countries (regions) in the field of intermittent fasting research according to the number of publications. The United States is the center of global cooperation in this field and cooperates most closely with other countries and regions due to the highest publications (424 articles, accounting for 38.34% of the literature sample). According to the theory of the Yuasa phenomenon (45), the country whose research results account for more than 25% of the total number of scientific results at a given time can be called the world center of science for that period, the United States, as the leading country in intermittent fasting research, has published more than a quarter of the total number of articles and is the world scientific center in the field of intermittent fasting. China (70), England (70), Australia (67) and Germany (55) followed closely in terms of volume. When analyzing in terms of centrality, the USA (0.49) was ranked first, followed by the European countries of Australia (0.15), Germany (0.13), Italy (0.12) and England (0.12). The high-centeredness country has an important place in the study of intermittent fasting, and it is worth noting that countries such as Italy, Germany and Australia, although the volume of articles is small, but the impact is large.

European and American countries have a high influence in this field, compared with China's 2nd place in the number of articles and 0.04 centrality, indicating that although China's ranking in the number of articles is at the top, it is less connected in the network map and has a lower influence. Therefore, increasing the depth of intermittent fasting research, accelerating multidisciplinary and multi-disciplinary cooperation, and enhancing the innovative thinking and international exchange ability of researchers are effective ways to improve research of intermittent fasting in China.

#### Distribution of Issuing Institutions

**Figure 4:**
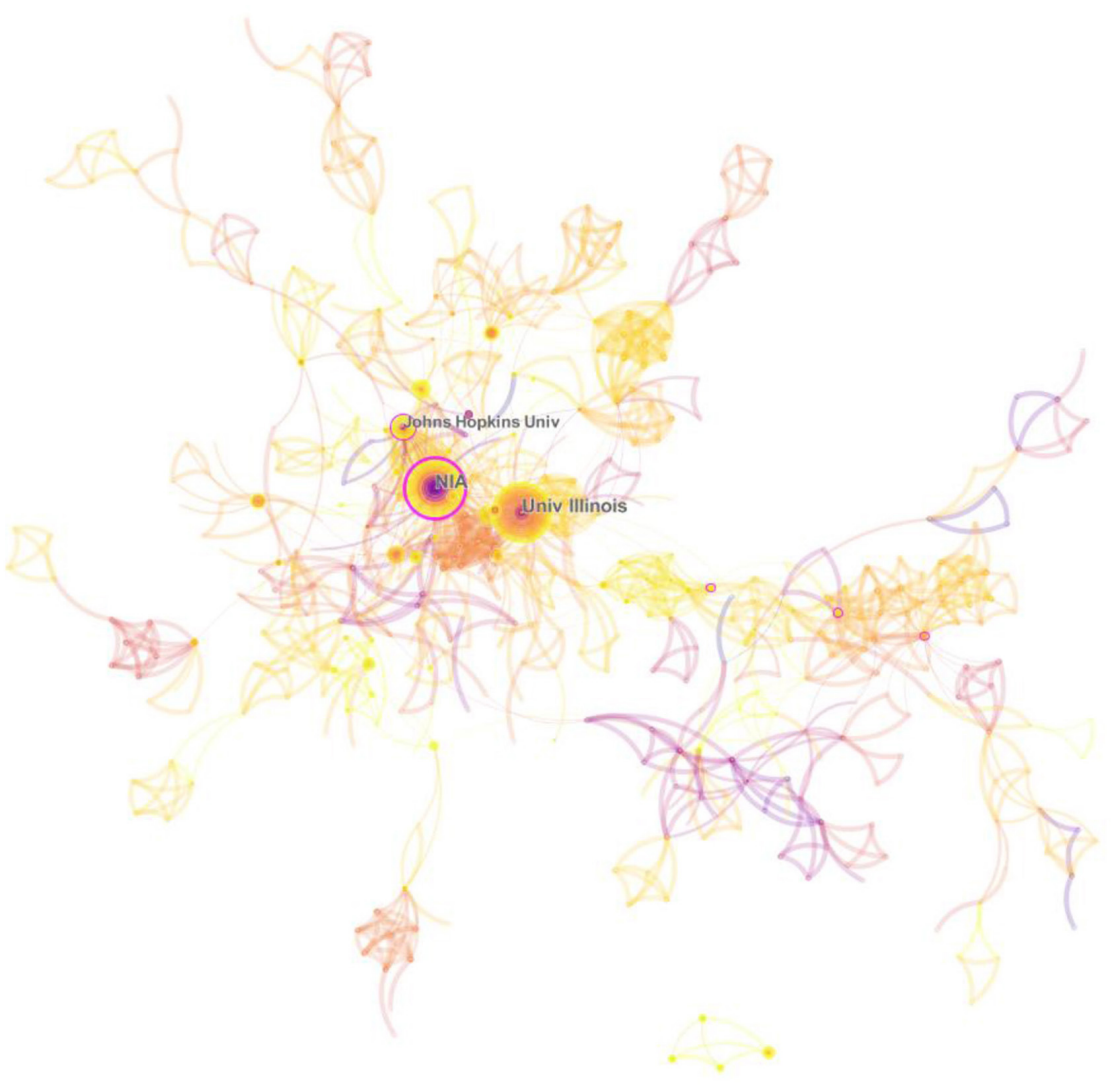
Knowledge map of co-institution collaboration network.

Figure 4 is a co-occurrence diagram of research institutions consisting of 813 nodes and 1,723 connecting lines. The nodes represent research institutions and the connecting lines between nodes reveal the cooperation between institutions; the thicker the line, the stronger the cooperation. It has many lines between nodes, indicating close cooperation between institutions.

Research institutions with a high frequency of publications are identified as influential institutions (46). Table 2 collates the top 15 research institutions in the field of intermittent fasting research in terms of the number of publications, which are the influential research institutions in the field of intermittent fasting. The results show that universities are the mainstay of intermittent fasting research. Among them, National Institute on Aging (NIA) has the highest number of articles (47), the highest centrality (0.22) and the most prominent impact. The United States accounted for 73.33% of the top 15 research institutions in terms of the number of articles published, further demonstrating its leadership role in the field of intermittent fasting research.

**Table 2:** Top 15 institutions in the field of intermittent fasting research in terms of number of publications.

**Institutions**	**Country of affiliation**	**Publications**	**Centrality**
NIA	United States	52	0.22
Univ Illinois	United States	51	0.08
Johns Hopkins Univ	United States	28	0.11
Univ São Paulo	United States	19	0.04
Texas Tech Univ	United States	18	0.09
Univ Alabama Birmingham	United States	18	0.03
Salk Inst Biol Studies	United States	18	0.08
King Saud Univ	Saudi Arabia	17	0.00
Univ Florida	United States	17	0.03
Univ Calif San Diego	United States	16	0.01
Univ Sydney	Australia	15	0.01
Univ Adelaide	Australia	14	0.01
Univ Toronto	Canada	13	0.11
Washington Univ	United States	13	0.03
Pennington Biomed Res Ctr	United States	13	0.01

### Discipline Analysis

**Figure 5:**
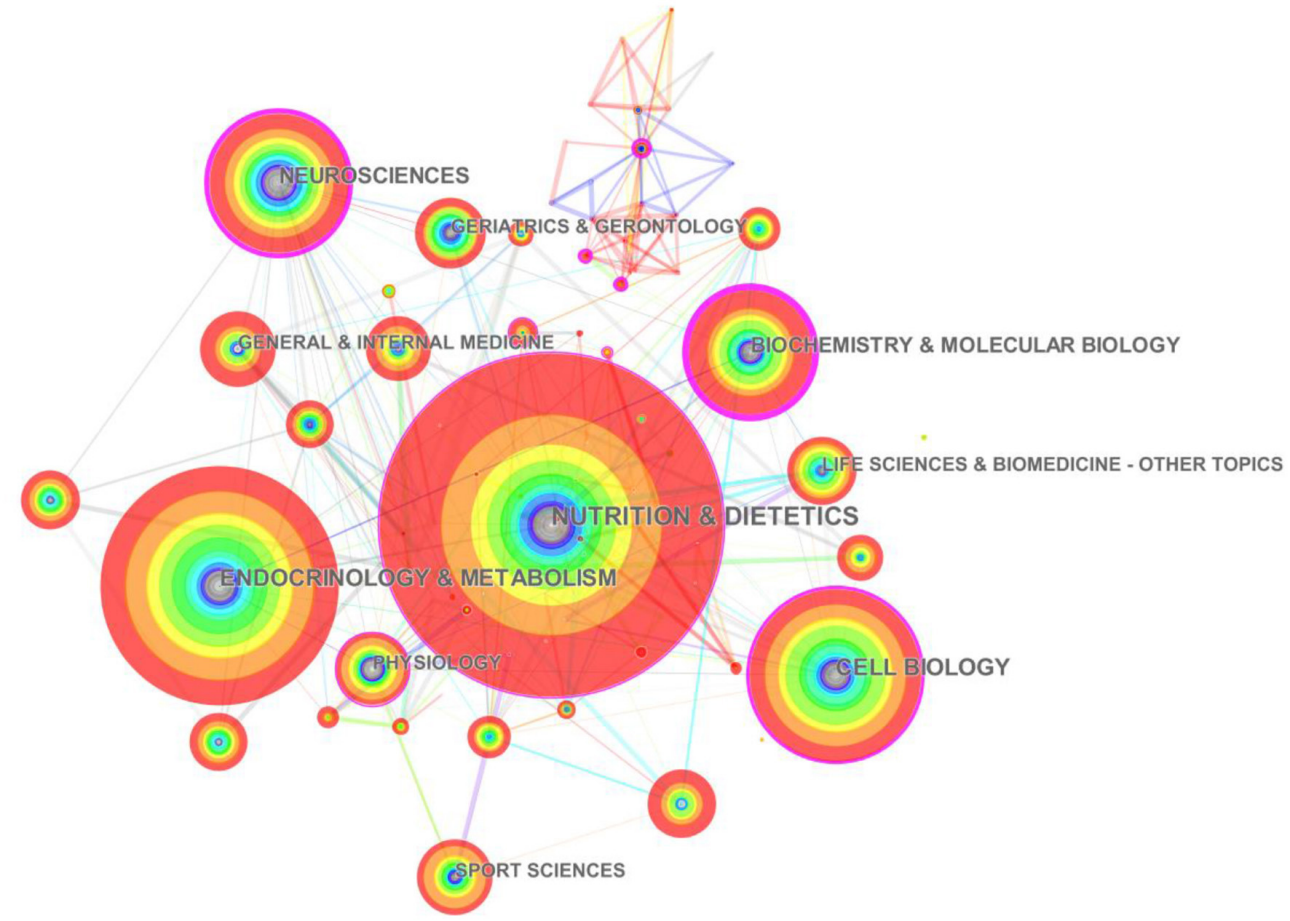
Knowledge mapping of disciplines related to intermittent fasting research.

By using the “Category” node of CiteSpace to perform disciplinary co-occurrence operations, we can obtain a knowledge map of disciplines related to intermittent fasting research (Figure 5). Knowledge mapping of disciplines may provide a first glimpse into the broad international classification of intermittent fasting in terms of disciplines. The order based on the frequency of discipline source are as follows: nutrition (262 times, centrality 0.14), endocrinology (184 times, centrality 0.08), cell biology (139 times, centrality 0.21), neurology (112 times, centrality 0.4), molecular biology (102 times, centrality 0.65), and sports science (67 times, centrality 0). The distribution of disciplines shows that intermittent fasting is a multidisciplinary field of study, combining the characteristics of multiple disciplines such as nutrition, biology and sports. At the same time, there has been a major trend toward the use of multidisciplinary cross-sectional trials for the study of intermittent fasting, which has also promoted further developments in this area.

### Highly Productive Paper Authors

**Figure 6:**
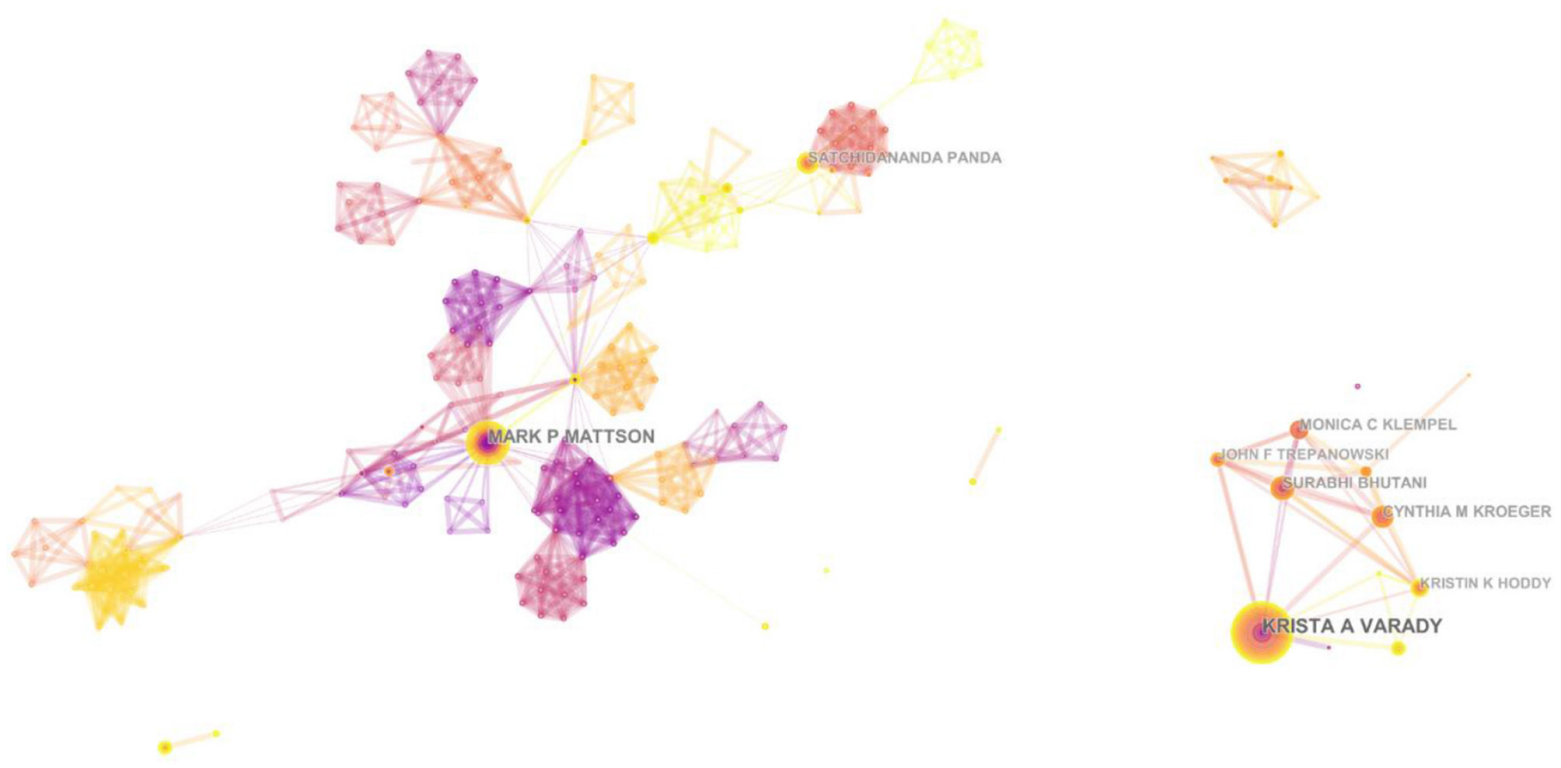
Knowledge map of co-author collaboration network.

Using CiteSpace software to analyze the co-author collaboration network, we can get the knowledge map of co-author collaboration network. As can be seen in Figure 6, the authorship of intermittent fasting research is relatively stable, with author groups usually containing two or more core authors. The field of intermittent fasting has formed a core group of academic teams such as VARADY and MATTSON respectively. Table 3 presents the top 10 authors in terms of the number of publications. Professor Varady has published 48 publications, ranking first and second in total citations (2,699), and his study included in Cell Metabolism in 2020 compared the effects of two popular forms of TRF (4 and 6 h) on weight and cardio metabolic risk factors and found restricted feeding promises to be an effective weight loss intervention (48). With MATTSON as the core team, the number of Publications (37) ranked second, H-index (49), the total number of citations(5,758)and average citations (156) ranked first. And the network of cooperation is dense, with a number of teams to form close cooperation.

**Table 3:** Top 10 authors in the field of intermittent fasting in terms of number of published articles.

**Rank**	**Author**	**Publications**	**H-index**	**Country**	**Total citations**	**Average citations**
1	KRISTA A VARADY	48	33	United States	2,699	56
2	MARK P MATTSON	37	68	United States	5,758	156
3	SURABHI BHUTANI	22	16	United States	1,681	76
4	SATCHIDANANDA PANDA	20	38	United States	2,468	123
5	CYNTHIA M KROEGER	20	11	Australia	849	42
6	MONICA C KLEMPEL	19	19	United States	1,045	55
7	KRISTIN K HODDY	17	2	United States	783	46
8	JOHN F TREPANOWSKI	15	22	United States	1029	69
9	GRANT M TINSLEY	14	18	United States	478	34
10	LEONIE K HEILBRONN	13	38	Australia	595	46

As a whole, the author collaboration map shows a large concentration and a small fragmentation, indicating a large collaboration pattern have been established, whereby these teams, as the key force of studying intermittent fasting, play an important role in advancing the development of this field.

### Journal Distribution

Analyzing the source journals of a certain research field can help researchers grasp the core journals of this field accurately and provide reliable reference sources for further research in this field (50). Table 4 shows the 10 journals with the most published articles. Among them, NUTRIENTS, PLOS ONE, CELL METAB, NUTRITION and ENDOCRINOLOGY are the top five journals. NUTRIENTS published the most articles, the second most total citations. The number of published articles in CELL METAB ranked third, but the Total Citations (3,964), Impact Factor (27.287) and CiteScore (40.7) ranked first. This phenomenon indicates that the academic papers published by CELL METAB are of high quality and have great influence.

**Table 4:** Top 10 journals with the most published articles.

**Journal**	**Total publications**	**Quartile score**	**Impact factor (2020)**	**Total citations**	**CiteScore (2021)**
NUTRIENTS	54	Q1	5.179	1,438	7.9
PLOS ONE	25	Q2	3.240	856	5.6
CELL METAB	17	Q1	27.287	3,964	40.7
NUTRITION	17	Q2	4.008	366	6.8
ENDOCRINOLOGY	16	Q2	4.736	1,399	8.4
METABOLISM	16	Q1	8.697	451	16.5
BRIT J NUTR	15	Q3	3.718	376	6.0
OBESITY	14	Q4	5.002	918	9.9
PHYSIOL BEHAV	14	Q2	3.244	307	5.7
AM J CLIN NUTR	13	Q1	7.047	1,317	10.6

### Literature Co-citation Analysis

**Figure 7:**
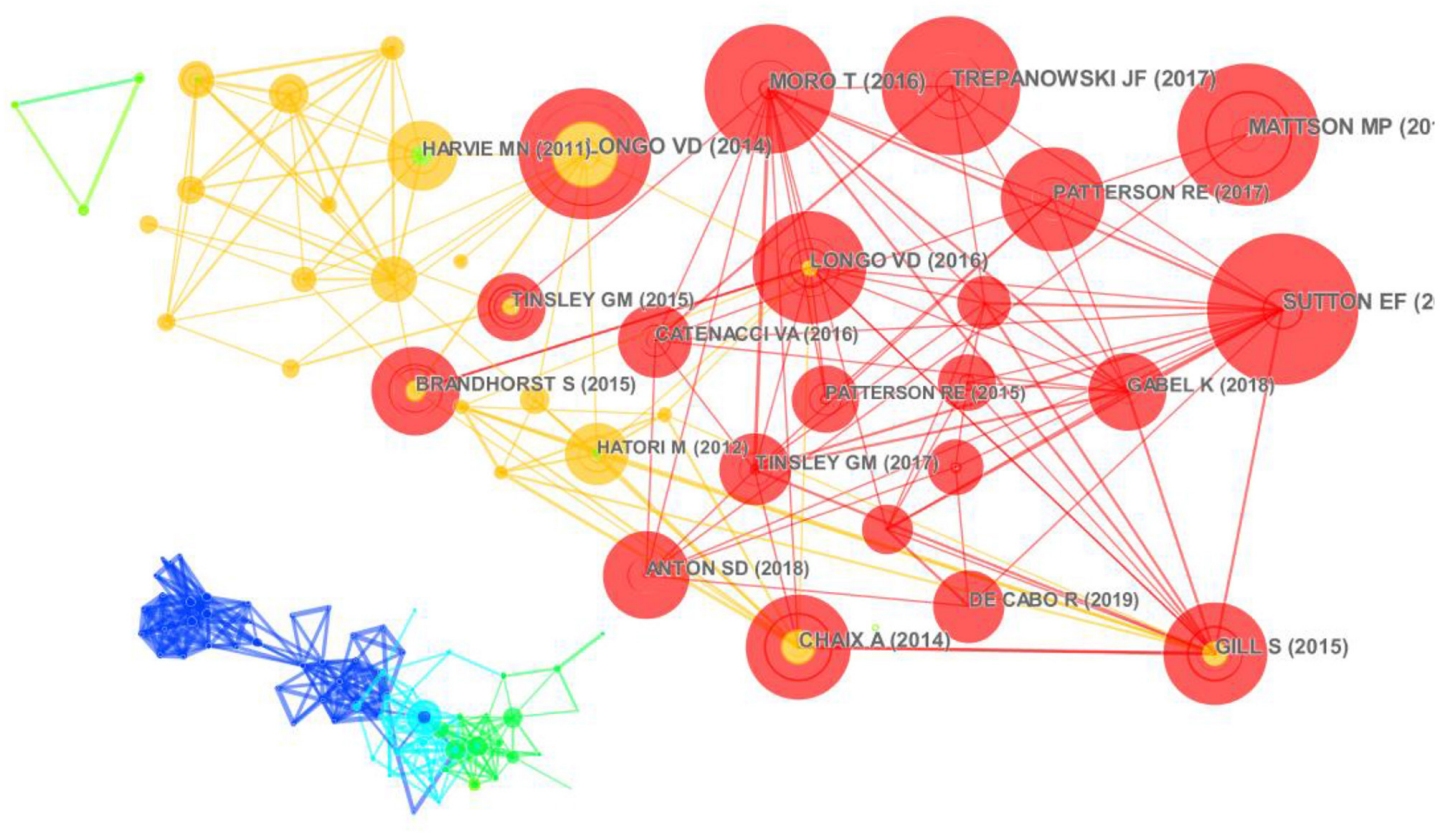
Co-citation analysis mapping of literature related to intermittent fasting.

Literature co-citations are used to assess the impact of literature. The time slice was set to 3 years, and the data of the top 20 citation frequency in each time slice were extracted, and there was a total of 339 nodes and 1,087 links. The size of the nodes is closely related to the frequency of citations, and the thickness of the line between the nodes indicates the closeness of the connection. As seen in Figure 7, Sutton et al. (15) had the largest literature node with 87 citations, ranking first. The researcher conducted a clinical trial of early time restricted feeding (e-TRF) on eight pre-diabetic men; the study found that when taking the same food intake as the control group, subjects did not lose weight but improved insulin levels, insulin sensitivity, suggesting that the benefits of time restricted feeding were not related to food intake and weight loss. The second most cited literature is the article published by Mattson et al. (24); this study provides a systematic review of the literature on fasting and indicates that time restricted feeding therapy has a positive impact on health indicators in both animals and humans, as well as improving many health indicators in healthy individuals and those with certain chronic conditions; in the meantime, this publication has been accepted for publication. The third cited literature is the article by Trepanowski et al. with a total of 79 citations (42), which stated that alternate-day fasting did not produce better results when compared to daily calorie restriction trials. Also, this trial was the longest lasting and largest trial. All three papers were published in the same phase and had high co-citation frequency, but the research perspectives were quite different, which reflects the different opinions of intermittent fasting. Therefore, IF field is still evolving.

### Literature Analysis

The top cited article was published in CELL METABOLISM in 2012 by Hatori, M, with a total of 762 citations. Among the top 10 papers with the most citations, the highest journal-level article was published in SCIENCE by Panda, S in 2016. Of the top 10 highly cited articles, four of them were published in the journal CELL METABOLISM, and there were four reviews and six articles in them (Table 5).

**Table 5:** Top 10 papers with the most citations.

**Rank**	**Title**	**Author**	**Type**	**Journal**	**Year**	**Citations**
1	Time-Restricted Feeding without Reducing Caloric Intake Prevents Metabolic Diseases in Mice Fed a High-Fat Diet	Hatori, M	Article	CELL METABOLISM	2012	762
2	Promoting Health and Longevity through Diet: From Model Organisms to Humans	Fontana, L	Review	CELL	2015	500
3	Intermittent Fasting Dissociates Beneficial Effects of Dietary Restriction on Glucose Metabolism and Neuronal Resistance to Injury From Calorie Intake	Anson, RM	Article	PROCEEDINGS OF THE NATIONAL ACADEMY OF SCIENCES OF THE UNITED STATES OF AMERICA	2003	394
4	Intermittent Fasting and Caloric Restriction Ameliorate Age-Related Behavioral Deficits in the Triple-Transgenic Mouse Model Of Alzheimer's Disease	Halagappa, VKM	Article	NEUROBIOLOGY OF DISEASE	2007	317
5	Time-Restricted Feeding Is a Preventative and Therapeutic Intervention Against Diverse Nutritional Challenges	Chaix, A	Article	CELL METABOLISM	2014	306
6	Beneficial Effects of Intermittent Fasting and Caloric Restriction on the Cardiovascular and Cerebrovascular Systems	Mattson, MP	Article	JOURNAL OF NUTRITIONAL BIOCHEMISTRY	2005	277
7	Circadian Physiology of Metabolism	Panda, S	Review	SCIENCE	2016	275
8	Diet and Feeding Pattern Affect the Diurnal Dynamics of the Gut Microbiome	Zarrinpar, A	Article	CELL METABOLISM	2014	271
9	Fasting, Circadian Rhythms, and Time-Restricted Feeding in Healthy Lifespan	Longo, VD	Review	CELL METABOLISM	2016	241
10	Impact of Intermittent Fasting on Health and Disease Processes	Mattson, MP	Review	AGING RESEARCH REVIEWS	2017	219

## Emerging Trends and Research Frontiers in Intermittent Fasting

### Emerging Trends

**Figure 8:**
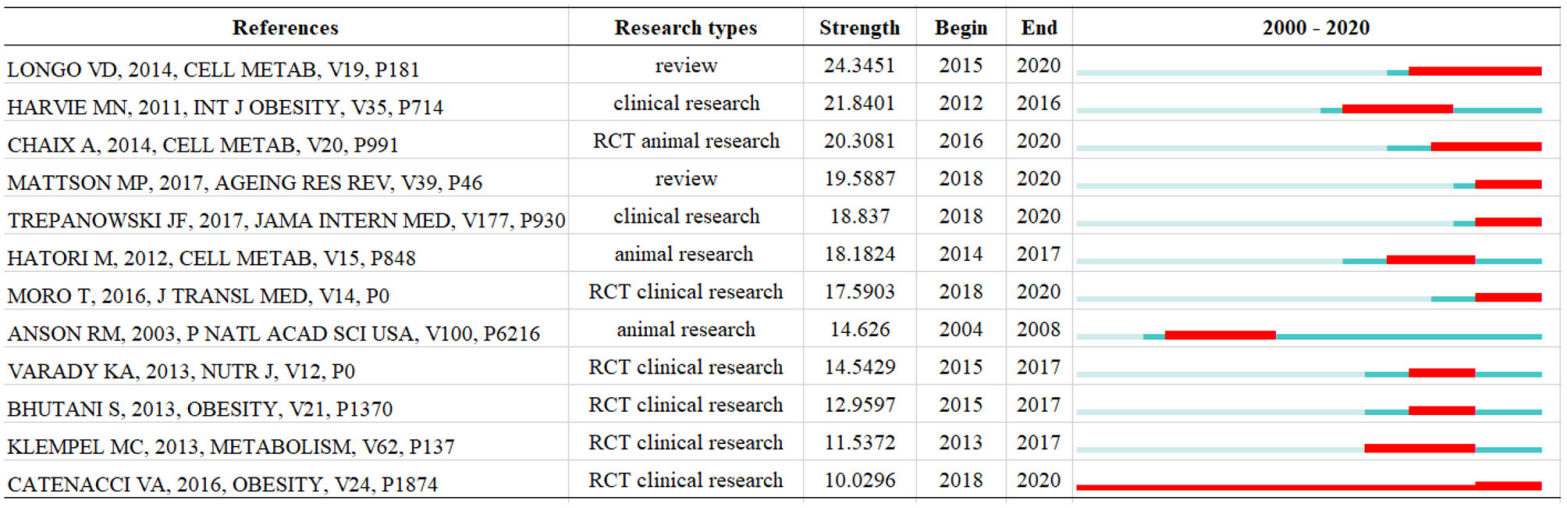
Top 12 references with the strongest citation bursts.

CiteSpace offers burst detection, which detects when relevant literature has attracted the relevant attention in the field for a certain period of time (51). References with high intensity values in the intensity column are often important milestones in scientific mapping research (52). Thus, the focus of the intermittent fasting research field can be found. The top 12 literature with the highest citation intensity from 2000 to 2020 are listed in Figure 8, highlighting the direction of focus and research trends in intermittent fasting over the last 20 years. Of these 12 articles, seven were clinical studies, three animal studies, and two reviews, most of which were RCT studies.

Since 2004, the field has focused mainly on caloric restriction research. Anson et al. (41) compared intermittent fasting with a caloric restriction protocol and found that intermittent fasting produced health benefits that outweighed caloric restriction. Subjects often spontaneously tend to eat less globally as there were fewer food opportunities. But a study found that intermittent fasting also had beneficial effects on glucose regulation and resistance to neuronal damage in mice, and these benefits were independent of calorie intake.

The trend in intermittent fasting research after 2013 shifted to studies of alternate day fasting. Varady et al. (53) conducted studies on humans and found that alternate-day fasting had a weight loss and cardioprotective effect in normal weight people, and that dietary adherence was high in this trial. Bhutani et al. (17) compared ADF combined with endurance training with ADF alone, an exercise intervention, and found that ADF combined with endurance training produced better changes in body weight, body composition and lipid indicators of coronary heart disease risk. Although ADF combined with a low-fat diet can be effective for weight loss and cardio protection. Given that most Americans consume a high-fat (HF) diet, Klempel et al. (47) explored the physical effects of consuming a high-fat diet during ADF and found that ADF, even in a high-fat setting, can also help obese people to lose weight and reduce the risk factors for coronary heart disease.

After 2018, intermittent fasting studies focused on ADF and TRF studies, which focused more on safety studies of fasting and more flexible forms of fasting, while, at the same time, began to experiment with studies in various populations. Longo et al. (12) noted that PF, TRF and IF have profound beneficial effects on rodent and human health. In rodents, intermittent or periodic fasting prevents diabetes, cancer, heart disease and neurodegeneration (41). In human studies, intermittent fasting has helped with obesity, hypertension, asthma and rheumatoid arthritis. Incorporating an IF or PF lifestyle into adult life has great potential to promote optimal health and reduce the risk of many chronic diseases, especially for those who are overweight and sedentary. Mattson et al. (24) reviewed the effects of intermittent fasting on health and disease processes and also confirmed the many benefits of intermittent fasting. Catenacci et al. (54) point out that alternate day eating is safe and tolerable and is a reasonable alternative dietary strategy for the treatment of obesity. However, Trepanowski et al. (42) compared alternate-day fasting with daily calorie-restricted diets and found that alternate-day fasting was not superior to daily calorie-restricted diets in terms of compliance, weight loss, weight maintenance or improvements in cardiovascular disease risk indicators. Chaix et al. (1) concluded that TRF has great potential in combating obesity and its associated metabolic disorders. Although TRF emphasizes the benefits of daily fasting cycles, the 5T2A approach (5 days of TRF combined with a two-day casual diet at weekends) is equally effective and the food intake of 5T2A is almost identical to that of the cycle, suggesting that TRF diets can resist occasional changes in eating patterns. That is, even occasional shifts in dietary patterns do not diminish the benefits of TRF. However, when mice transiting from TRF to free-feeding mode, the benefits of TRF were gradually diminished. Conversely, mice with gradual transition from free-feeding to a time restricted feeding pattern can effectively alleviate the adverse health conditions associated with obesity. The results of this study suggest that TRF interventions have a positive effect on bodybuilder performance and athletes can adopt this diet during the maintenance phase of training, which facilitates the reduction of fat mass while maintaining muscle mass (55).

The focus of research in this area over the last 20 years is roughly divided into three phases in terms of the 12 most highly cited papers: Phase 1 (2004–2008), which focused on the study of caloric restriction for human health. In the second phase (2013–2017), the trend in intermittent fasting research shifted to research on alternate-day fasting. In the third phase (2018–2020), the focus in this period was on alternate-day fasting and time restricted feeding, but the difference is that the research in this period went deeper and began to compare the compliance, safety and health benefits of dietary practices, thus exploring a better way of eating to serve the health of diverse populations.

### Frontiers of Research

**Figure 9:**
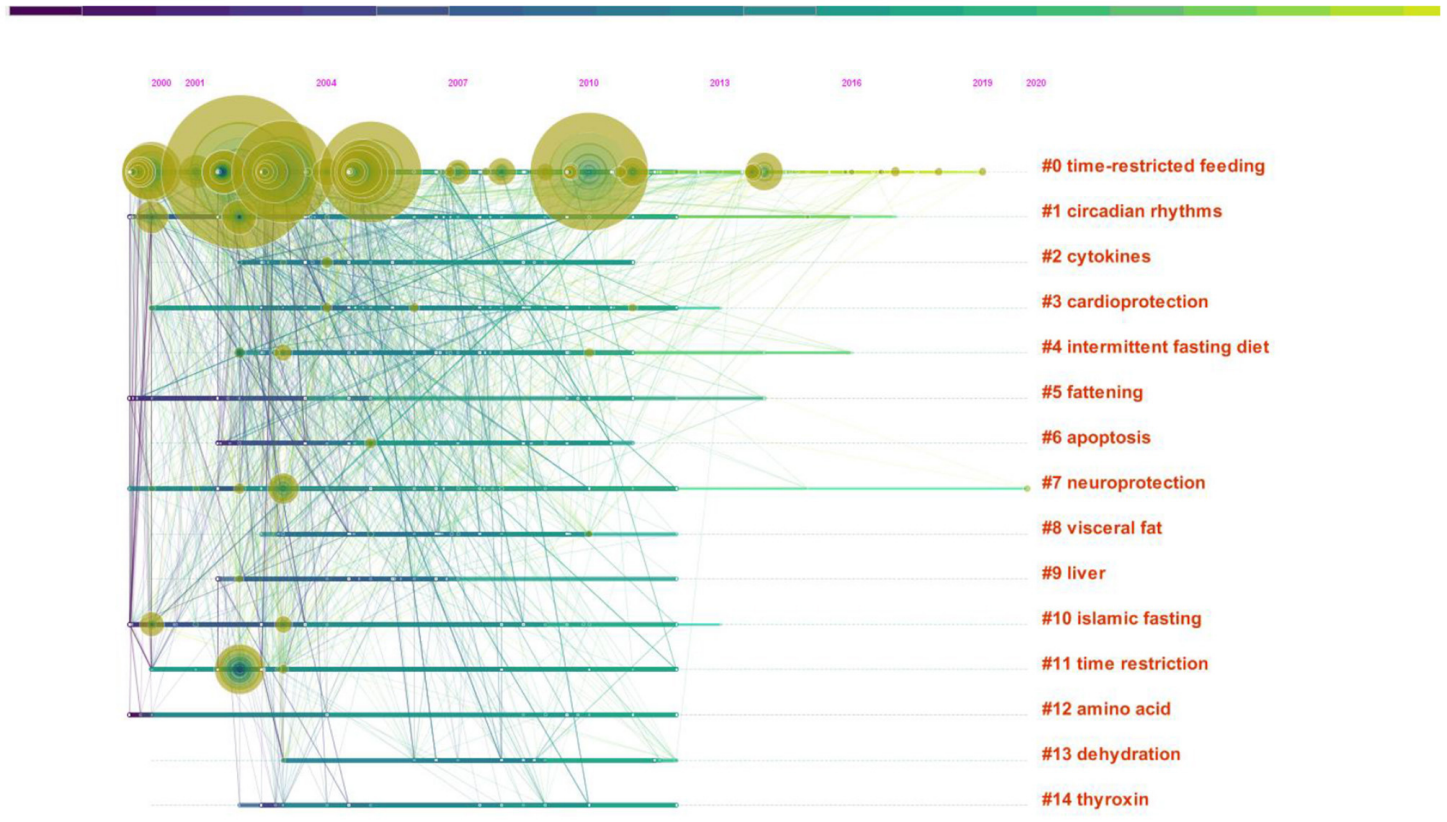
Time-line mapping of keywords in the field of intermittent fasting research 2000-2020.

The timeline view focuses on reflecting the relationships between clusters and the historical span of the literature in a given cluster, and also reflects the timing of distribution and connection of key keywords between clusters (56, 57). Figure 9 shows the timeline mapping of keywords in the field of intermittent fasting research from 2000–2020. The key parameters of clustering are Modularity Q value of 0.6,512 > 0.3 and S-value of 0.5,675 > 0.5, which can indicate the formation of reasonable clusters (58). The figure shows that time restricted feeding has been active since 2000 and is closely linked to other clusters and is at the forefront of research in this area. Time restricted feeding is a non-pharmacological technique for obesity and related diseases (2), which has been shown to have a significant effect on improved quality of life (59). Time restricted feeding is mainly used to achieve weight loss by reducing appetite (60). Besides preventing obesity and improving metabolic disease (61–65), time restricted feeding can improve blood sugar, lipid metabolism, and chronic diseases (49, 66–68). It is worth noting that in the restricted eating state, the benefits of 2 days of random eating in a week in subjects are also beneficial to the organism (2), and time restricted eating is beneficial to human health even in the absence of weight loss (69).

## Conclusions

On the basis of 11,066 intermittent fasting studies from WOS from 2000 to 2020, CiteSpace was utilized to visually analyze the volume of articles published, distribution characteristics (country/region, institution), collaborations (country/region, institution, author), literature co-citations, and the frontiers of intermittent fasting research and trends. The main findings of this study are as follows:

Firstly, the number of articles in the field of intermittent fasting is increasing year by year. From the graph of the temporal distribution, intermittent fasting studies can be divided into two phases: phase 1 is the steady growth phase (2000–2016), during this period, the number of publications is slowly increasing; phase 2 is the continuous growth phase (2017–2020), in which there was a surge in the number of publications with an average of 167 publications per year, indicating the popularity and importance of intermittent fasting. This trend is also supported by the extensive literature on intermittent fasting since 2021.

Secondly, the United States has significantly higher publication volume and more influence than other countries, and maintains close cooperation with other countries and regions, indicating that the United States plays an important role in IF field. Although China ranks 2nd in the number of publications, however, the level of academic collaboration in this field is much lower than that of other countries; therefore, cooperation between international institutions needs to be strengthened.

Thirdly, intermittent fasting is typically an interdisciplinary study, numerous disciplinary categories are involved, mainly covering the disciplines of nutrition, biology and kinesiology. Most of the scholars who have participated and made outstanding contributions to intermittent fasting research have a medical background. For example, the representative American scholar Professor Mattson is at the National Institutes of Health. However, there is no shortage of scholars with a sports background, such as Professor Varady, a faculty member in the Department of Exercise and Nutrition at the University of Illinois, who is the most published scholar in the field of intermittent fasting and has made outstanding contributions to this field.

Fourthly, intermittent fasting has a variety of modalities and the impact of applying this dietary strategy has been inconsistent. Intermittent fasting has received considerable attention in the past few years, but its safety and compliance has been questioned (70, 71). However, the use of intermittent fasting in China has been viewed with caution. Most of the studies on intermittent fasting in China are literature reviews and animal studies.

Fifthly, the focus of intermittent fasting research has gone through three phases over the past two decades: caloric restriction, alternate-day eating, and time restricted eating. Time-restricted eating has been active for two decades and is the focus of current research in this field, which is strongly linked to the promotion of physical health.

In 2020, Professor Wilkinson found that TRE interventions, which do not require attempts to change physical activity or diet, can also have a positive effect on the treatment of metabolic syndrome (3). Professor Ulgherait in the journal NATURE in 2021. It suggests that TRF may have benefits in delaying aging and prolonging life (72). A 2022 study by Professor Mao YL showed that TRF not only improves fasting blood glucose and inflammation, but also increases intestinal microbial diversity (73) and be expected to become health-promoting lifestyle (48). The above studies suggest that the culmination of research on time restricted eating in intermittent fasting is imminent.

The field of intermittent fasting research has achieved a rapid growth over the past 20 years, and the growing literature on intermittent fasting each year indicates that the field has become a research hotspot in recent years. The interdisciplinary research context of intermittent fasting will facilitate international cooperation among scholars to find more scientific, non-pharmacological techniques. The penetration of intermittent fasting into people's health may change our existing lifestyle. Time restricted eating does not restrict caloric intake, therefore, it can become an easily accepted and healthy lifestyle.

## Data Availability Statement

The original contributions presented in the study are included in the article/supplementary material, further inquiries can be directed to the corresponding author/s.

## Author Contributions

HL was responsible for concept, design, and supervision. SC collected data from the database and wrote the original draft preparation. HL and RH took the responsibility of critical revision. All authors contributed to the article and approved the submitted version.

## Funding

This work was supported by Henan Science and Technology Development Project grants (202102310320), Henan Science and Technology Development Project grants (212102310260), Henan Education Science Planning Project grants (2021YB0031), Henan Higher Education Institutions Project grants (20A890003).

## Conflict of Interest

The authors declare that the research was conducted in the absence of any commercial or financial relationships that could be construed as a potential conflict of interest.

## Publisher's Note

All claims expressed in this article are solely those of the authors and do not necessarily represent those of their affiliated organizations, or those of the publisher, the editors and the reviewers. Any product that may be evaluated in this article, or claim that may be made by its manufacturer, is not guaranteed or endorsed by the publisher.
